# Polarimetry with Spins in the Solid State

**DOI:** 10.1021/acs.nanolett.5c01511

**Published:** 2025-05-07

**Authors:** Lorenzo Peri, Felix-Ekkehard von Horstig, Sylvain Barraud, Christopher J. B. Ford, Mónica Benito, M. Fernando Gonzalez-Zalba

**Affiliations:** † 151956 Quantum Motion, 9 Sterling Way, London, N7 9HJ, United Kingdom; ‡ Cavendish Laboratory, 646252 University of Cambridge, JJ Thomson Ave, Cambridge CB3 0HE, United Kingdom; § Department of Materials Sciences and Metallurgy, 173532 University of Cambridge, Charles Babbage Rd, Cambridge CB3 0FS, United Kingdom; ∥ 2152 CEA, LETI, Minatec Campus, F-38054 Grenoble, France; ⊥ Institute of Physics, University of Augsburg, Augsburg, 86159, Germany; $ CIC nanoGUNE BRTA, 20018 Donostia-San Sebastian, Basque Country, Spain; % IKERBASQUE, Basque Foundation for Science, 48013 Bilbao, Basque Country, Spain

**Keywords:** Spins, Spin Qubits, Pauli Spin Blockade, Spin−Orbit Coupling, Quantum Dots, Polarimetry

## Abstract

The ability for optically active media to rotate the polarization of light is the basis of polarimetry, a prominent technique responsible for many breakthroughs in fields as varied as astronomy, medicine, and material science. Here, we recast the primary mechanism for spin readout in semiconductor-based quantum computers, Pauli spin-blockade (PSB), as the natural extension of polarimetry to the third dimension. We perform polarimetry with spins through a silicon quantum dot exchanging a hole with a boron acceptor, demonstrating the role of spin–orbit coupling in creating spin misalignment. Perfect spin alignment may be recovered by means of rotating the applied magnetic-field orientation. This work shows how spin misalignment sets a fundamental upper limit for the spin readout fidelity in quantum-computing systems based on PSB.

## Graphical abstract

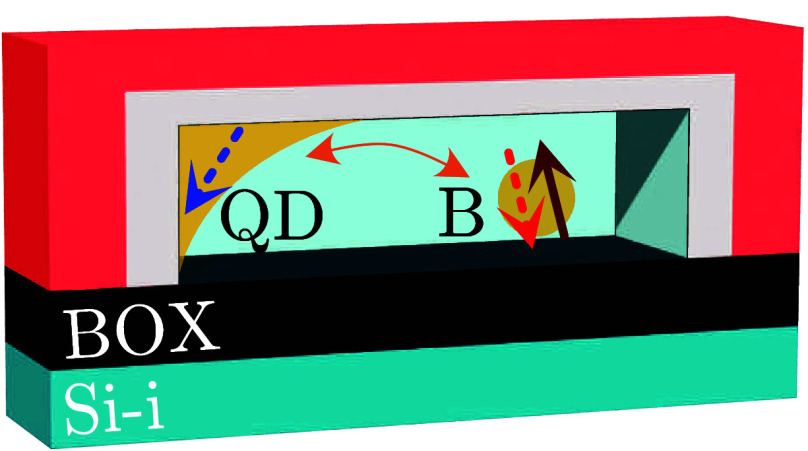

Ever since its introduction in 1834 by William Fox Talbot,^1–3^ polarimetry has been one of the main techniques for investigating the world around us.^4^ From tissues^5^ to stars,^6^ from radars and navigation^7–11^ to meteorology and industrial processes,^12,13^ from elucidating the nature of electromagnetic waves^14^ to understanding the behavior of chemical bonds,^15^ polarimetry has provided an unprecedented eye into the microscopic properties of matter and their interaction with a rotationally quantized property, such as the polarization of light.^16,17^

*In essentia*, the idea of polarimetry stems from the observation that particular degrees of freedom are *polarized* (i.e., quantized along an axis in orthogonal states), and that certain physical systems are *active*, lacking the relevant symmetries and allowing transitions between such states^18^ ^,^ [Fn fn1]. A breakthrough from this original view by George Gabriel Stokes^19^ was introduced by Jones and later by Mueller,^20^ who posited that (optical) *activity* is best represented as a *rotation* of the polarization (i.e., quantization) axis, plus a potential additional phase.^21^ This intuition provides the basis of the polarimeter (Figure 1(a)), in which an active medium is placed between two polarizing elements (one moveable, the *polarizer*, and one fixed, the *analyzer*). The optical transmission is measured as a function of polarizer angle, which quantifies the polarization misalignment between the analyzer and the image of the polarizer after rotation by the medium.

**Figure 1:**
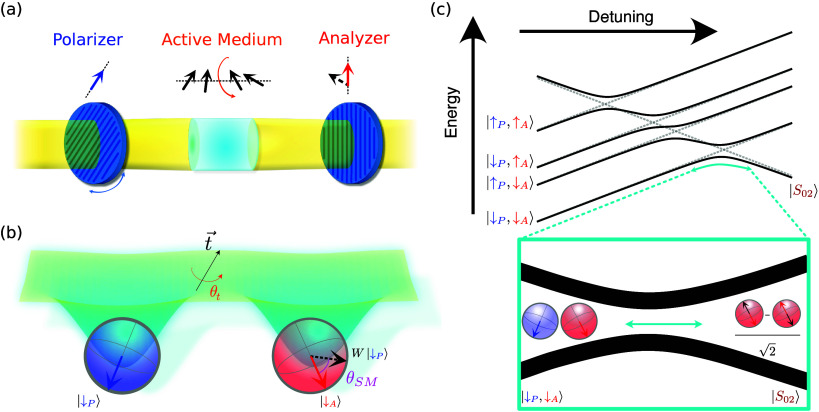
**Polarimetry with light and with spins.** (a) A standard light polarimeter. An active medium (capable of rotating the polarization axis) is placed between two polarizing elements (*polarizer* and *analyzer*) and the polarization misalignment angle is measured. (b) A spin–orbit-coupled double quantum dot seen as a polarimeter for spins. The two sites have different quantization axes, and spin–orbit coupling causes a spin rotation upon charge tunneling. (c) Energy diagram of an even-charge transition between (1,1) and (0,2) occupation. Spin–orbit coupling causes the crossing between the (1,1) triplet and the (0,2) singlet to be avoided (inset), lifting Pauli spin blockade.

In this work, we extend the concept of polarimetry to another physical system of technological interest possessing a polarized degree of freedom: spins confined in semiconductor quantum dots (QDs). We demonstrate how this platform, a promising candidate for quantum information processing,^22–26^ represents the natural extension of light polarimetry to the third dimension, and how concepts borrowed from the field of polarimetry provide valuable insight into the complex physics of coupled spins in the solid state, particularly when subject to spin–orbit coupling (SOC). Here, we exploit notions from polarimetry to provide a geometrical intuition for the effect of SOC on spin qubits, introducing the concept of the spin misalignment angle between two QDs (acting as analyzer and polarizer). We experimentally demonstrate our model by performing polarimetry of a hole spin exchanged between a silicon QD and a boron acceptor. Exploring different orientations of the applied magnetic field (polarizer direction), we measure the resulting spin misalignment, which vanishes at one particular *magic angle*,^27,28^ where the (rotated) image of the polarizer perfectly projects onto the analyzer. This corresponds to the physical phenomenon of Pauli spin-blockade (PSB), and it is the direct equivalent of perfect transmission through a light polarimeter.

For the purpose of quantum computation, SOC is attracting growing interest^29–33^ thanks to the opportunity of all-electrical universal qubit control.^34–36^ Yet, it inherently introduces additional challenges. The presence of SOC-induced spin misalignment manifests itself as lifting of the PSB, undermining the effectiveness of the spin-to-charge conversion (SCC) used by many spin-readout schemes.^28,29,37–39^ The analogy with polarimetry naturally leads us to introduce the concept of SCC fidelity to quantify such errors in the SCC process. This effect is notably missing from the spin-qubit literature, and mandates a necessary correction (and sets a fundamental upper limit) for the fidelity of PSB-based spin readout, which, given more than two readout sites, may globally be below the fault-tolerance threshold for error correction. This poses serious challenges for the scalability of spin-qubit architectures for quantum information processing, particularly those based on holes, which rely on Zeeman gradients as a control mechanism.^29,32,33,35^

The presence of SOC in a double QD manifests itself in two ways. First, it causes two spatially separated spins to experience the magnetic field differently. This is encoded in different *g*-tensors **g** for the two QDs. This effectively creates two separate (and generally misaligned) quantization directions **g** *B⃗* (known as *internal* fields) onto which each spin aligns when an *external* magnetic field *B⃗* is applied. However, SOC also allows interdot tunneling to mix spin states. Namely, tunneling may either *conserve* or *flip* the spin orientation. The former process can be described by a scalar *t*_0_, while the latter is not rotationally invariant and thus is most generally represented as a (real) vector *t⃗*.^28^ A key observation is that tunneling is not akin to a measurement that must produce a spin eigenstate (probabilistically *either* conserving *or* flipping the spin orientation). Rather, a tunneling process in the presence of SOC is best understood as accompanied by a unitary spin-axis *rotation* around the *t⃗* vector of an angle θ_*t*_/2 = (|*t⃗*|/*t*_0_), represented by the unitary transformation $W=\exp\left(\frac{\theta_{t}}{2i}\frac{\mathrm{t⃗}}{|\overset{⃗}{t|}}\cdot \mathrm{\sigma ⃗}\right)$ .^27^ Therefore, spins in the two QDs live on Bloch spheres with axes (mis)­aligned along their internal fields, and can only *see* each other through the *warped* lens of a spin precession.

Undoubtedly, the reader will be reminded of the previous discussion of polarimetry, only raised one dimension higher. This is understood by the fact that, unlike a *flat* polarizer rotating around a fixed axis, *g*-tensors are *three-dimensional*, and one may orient *B⃗* in any direction in 3D space.

The analogy with polarimetry is particularly suited to describing even-charge interdot transitions (i.e., (1,1) ↔ (0,2) charge occupation), owing to the Pauli exclusion principle. Spins occupying separate QDs will generally align with the internal fields and will therefore rotate as we modify the external *B⃗*. Conversely, when two spins occupy the same QD, the Pauli principle requires that they be in a singlet state (|*S*_02_⟩ = (|↑↓ – |↓↑⟩)/√2), which is a scalar under rotations, thus providing a *fixed reference* onto which to project. In keeping with the traditional polarimetry nomenclature, we henceforth refer to the QD that may host two spins as the (fixed) *analyzer* (red), and to the other as the (changeable) *polarizer* (blue).

A general treatment of the (1,1) ↔ (0,2) transition requires at least five states: the four spin orientations in the (1,1) occupation and |*S*_⟩_ (see Figure 1(c) and Supporting Information 1). However, in the case of a sufficiently high magnetic field—the exclusive focus of this work—the lowest energy state consists of the two spins antialigned with their respective internal fields (|↓_P_, ↓_A_⟩). Thereby, the low-energy dynamics can be simplified by considering the effective two-level Hamiltonian

1 $$H=\frac{1}{2}\left[\begin{array}{cc} \varepsilon -\mu_{B}\left(\left|g_{P}\mathrm{B⃗}\right|+\left|g_{A}\mathrm{B⃗}\right|\right) & \sqrt{2}\Lambda {\mathrm{t̃}}_{c}\\ \sqrt{2}\Lambda^{*}{\mathrm{t̃}}_{c} & -\varepsilon \end{array}\right]$$

where *ε* is the QD-boron energy detuning, ${\mathrm{t̃}}_{c}=\sqrt{t_{0}^{2}+|\mathrm{t⃗}{|}^{2}}$ is the total (zero-field) tunnel coupling, and

2 $$\left|\Lambda \right|=\left|\left\langle {↑}_{A}\right|W\right|{↓}_{P}\rangle |=\sin\frac{\theta_{\mathrm{SM}}}{2}$$

quantifies the *spin misalignment* of the (*t⃗*-rotated) polarizer state over the analyzer. Particularly, |Λ|^2^ represents the probability of a spin-flip upon projection of the rotated image of the polarizer onto the analyzer’s quantization axis. The complex spin dynamics can thus be summarized in the parameter |Λ| (or θ_SM_), which determines the high-field avoided crossing between the (1,1) and (0,2) ground states. Notably, for finite *t⃗*, the spin misalignment θ_SM_ is *not* the angle between the internal fields, as the latter fails to account for the *active* rotation induced by tunnelling in the presence of SOC. A more direct physical understanding is gained from observing that

3 $$\cos\theta_{\mathrm{SM}}=\frac{\left({\mathrm{Rg}}_{P}\mathrm{B⃗}\right)\cdot \left(g_{A}\mathrm{B⃗}\right)}{\left|g_{P}\mathrm{B⃗}∥g_{A}\mathrm{B⃗}\right|}$$

where **R** is the orthogonal matrix that represents the same rotation as *W* in 3D space.^27,28^ Equation 3 provides a geometrical interpretation of θ_SM_ as the angle of misalignment between the internal field of the analyzer and the image of the polarizer as seen through the active medium. Any reader well-versed in Lie algebras will not have missed the factor-of-two difference between the definitions of θ_SM_ in *SO*(3) (eq 3) and its double-covering *SU*(2) (eq 2), originating from antiparallel spins being *orthogonal* on the Bloch sphere.[Fn fn2].

We engineer our spin polarimeter in a solid-state system subject to strong SOC: a p-type single-gate silicon nanowire transistor. In particular, we study a system composed of an electrostatically defined QD underneath the gate of the transistor (polarizer) coupled to a boron acceptor in the channel (analyzer),^43^ see Figure 2a. From a measurement perspective, a key observation from eq 1 is that changes in Λ directly correspond to modifications of the |↓_P_, ↓_A_⟩ ↔ |*S*_02_⟩ anticrossing (Δ_SO_ = √2 |Λ|*t̃*_c_), and thus to the transition’s quantum capacitance. This parameter quantifies the system’s ability to tunnel between the two coupled states, and is thus directly related to Δ_SO_.^44^ We probe the quantum capacitance by the dispersive interaction with a high-*Q* superconducting microwave resonator (ω_r_/2π = 2.1 GHz) connected to the gate of the transistor. Specifically, changes in reflected power from the resonator reflect changes in the quantum capacitance, allowing a direct measure of |Λ|.^45^

**Figure 2:**
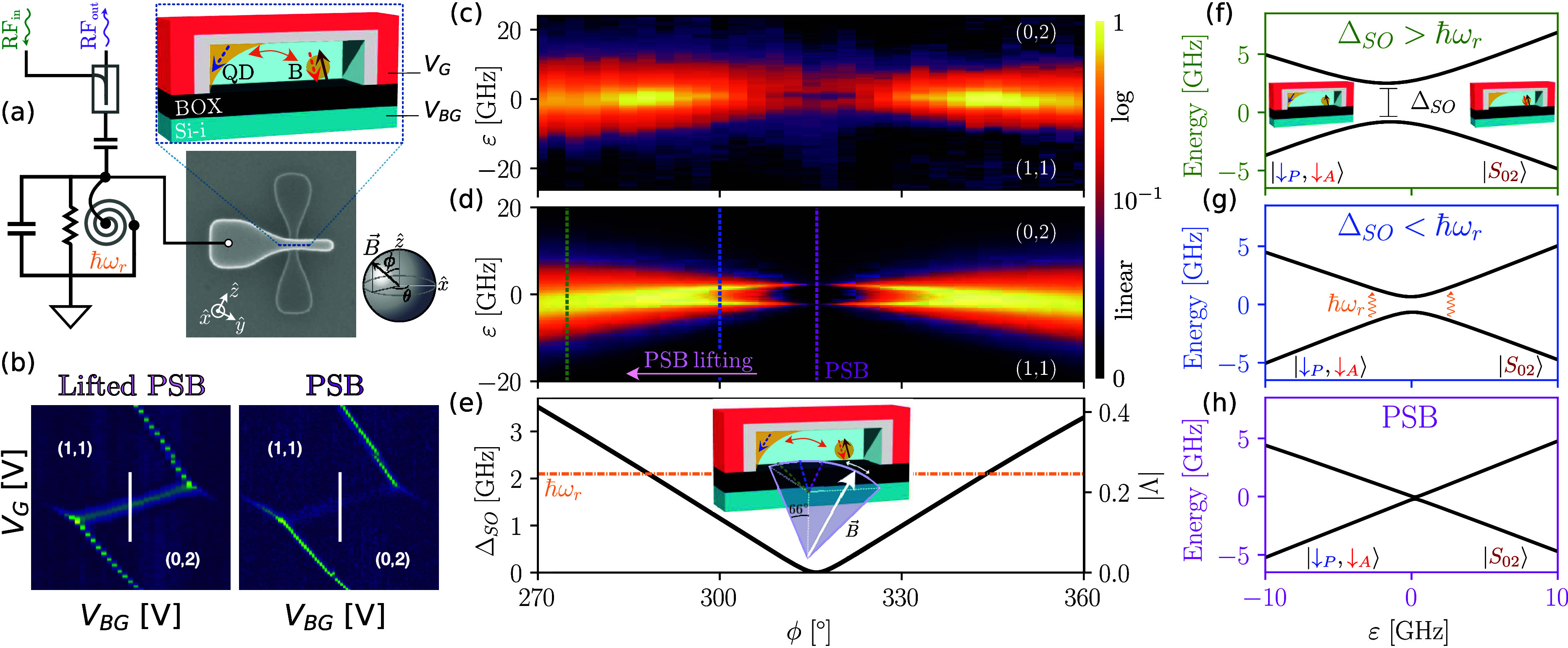
**A polarimeter for spins.** (a) A p-type silicon nanowire transistor hosting a spin–orbit-coupled system composed of an electrostatically defined QD and a boron acceptor (B). Micrograph of the device coupled to a microwave resonator (ω_r_/2π = 2.1 GHz), measured by reflectometry. The frame of reference for the magnetic field *B⃗* is the same as for the vector magnet in the dilution refrigerator. (b) Charge-stability map measured at high applied external field (|*B⃗*| = 0.5 T) of the (nominal) (1,1) ↔ (0,2) charge transition, in directions showing PSB (right) and its lifting (left). In the (0,2) charge state the spins reside in the boron acceptor. (c-e) Dispersive sensing of the transition for changing magnetic-field angle (data in (c) and theory in (d)) showing PSB and resonant splitting, and (e) fitted spin-misalignment parameter |Λ| (right axis) and spin–orbit gap (Δ_SO_ = √2*t̃*_c_ |Λ|) (left axis). (f-h) Energy diagrams of the spin–orbit anticrossing for various spin alignments.

We study a charge transition with nominal charge occupation (1,1) ↔ (0,2), (Figure 2b) measured in the high-field limit (|*B⃗*| = 0.5 T). The choice to have the boron as the doubly occupied QD avoids concerns about orbital, valley and higher spin-number states at high magnetic fields.^38,43^ The presence of signal at the interdot charge transition (ICT) indicates the ability to tunnel between the two charge (and spin) states |↓_P_, ↓_A_⟩ ↔ |*S*_02_⟩. However, at a specific orientation of the magnetic field (the *magic angle* where θ_SM_ = 0) the signal vanishes as tunneling becomes forbidden by PSB (Figure 2b).

We study this PSB transition experimentally and theoretically in Figure 2c,d by sweeping the in-plane magnetic-field orientation, ϕ, which includes the direction where we have preemptively located PSB. The experiment represents the spin equivalent of rotating a polarimeter’s polarizer to find the angle where light gets fully transmitted (an aligned light polarimeter is maximally *bright* while for spins it is maximally *dark* as the dispersive signal vanishes). As we rotate the field, we modify the spin misalignment, resulting in three distinct regimes: (i) lifting of PSB, (ii) resonant and (iii) PSB, whose energy diagrams along the respective green, blue and pink lines are represented in Figure 2f-h.

In the PSB-lifting regime (green line), the spins are misaligned, and the strong SOC in the system manifests as a single and broad peak centered at *ε* = μ_B_ (|**g**_P_ *B⃗*|+ |**g**_A_ *B⃗*|)/2.^43–46^ As we reduce Δ_SO_, the peak increases in magnitude and sharpens, until Δ_SO_ reaches the resonator frequency. Past that point, we reach the resonant regime (blue line), when Δ_SO_ ≤ ℏω_r_. In this intermediate region, we observe the signature of the resonant interaction between the QD-acceptor system and the photon cavity,^47–49^ which can be seen as incoherent spin rotations driven by the resonator.^50–52^ Finally, as the system approaches the *magic angle* (pink line), PSB is recovered and the dispersive signal vanishes as the image of the polarizer through the SOC medium perfectly aligns with the analyzer (θ_SM_ = 0). In this direction, the |↓_P_, ↓_A_⟩-|*S*_02_⟩ crossing is no longer avoided and spin and charge degrees of freedom remain uncoupled, leading to a vanishing dispersive signal and the loss of ability to drive the spins via the resonator. The electrical response of the system as the probe signal goes from adiabatic (green dashed line) to resonant (blue dashed line) can be described via our unified linear response theory^45^ (see Figure 2c and Supporting Information 2).

Unlike traditional light polarimetry, polarimetry with spins is intrinsically three-dimensional in nature, thus requiring characterization of the system as a function of both azimuth and zenith. Time-reversal symmetry guarantees the response to be equal for inversion of the field (*B⃗* → – *B⃗*),^28,34^ allowing the study to be limited to a solid angle of 2π. We do so exploiting our earlier observation that the dispersive signal can be used as a proxy for spin misalignment. Namely, the maximum signal will be small but finite for large Λ, peaking at the resonance condition Δ_SO_ = ℏω_r_ and then vanishing as the system’s dipole vanishes when the crossing is no longer avoided. In Figure 3a, we show that this trend appears as we approach the blockade from any direction, giving rise to a distinct ring-shaped region of intense signal encircling the PSB direction, which we name *PSB halo*. This halo occurs at a zenith angle θ_SM_ = 2 arcsin­(ℏω_r_/√2*t̃*_c_), describing a circle in the space of internal fields, and is then *warped* in Cartesian coordinates by *g*-tensor anisotropies.

**Figure 3:**
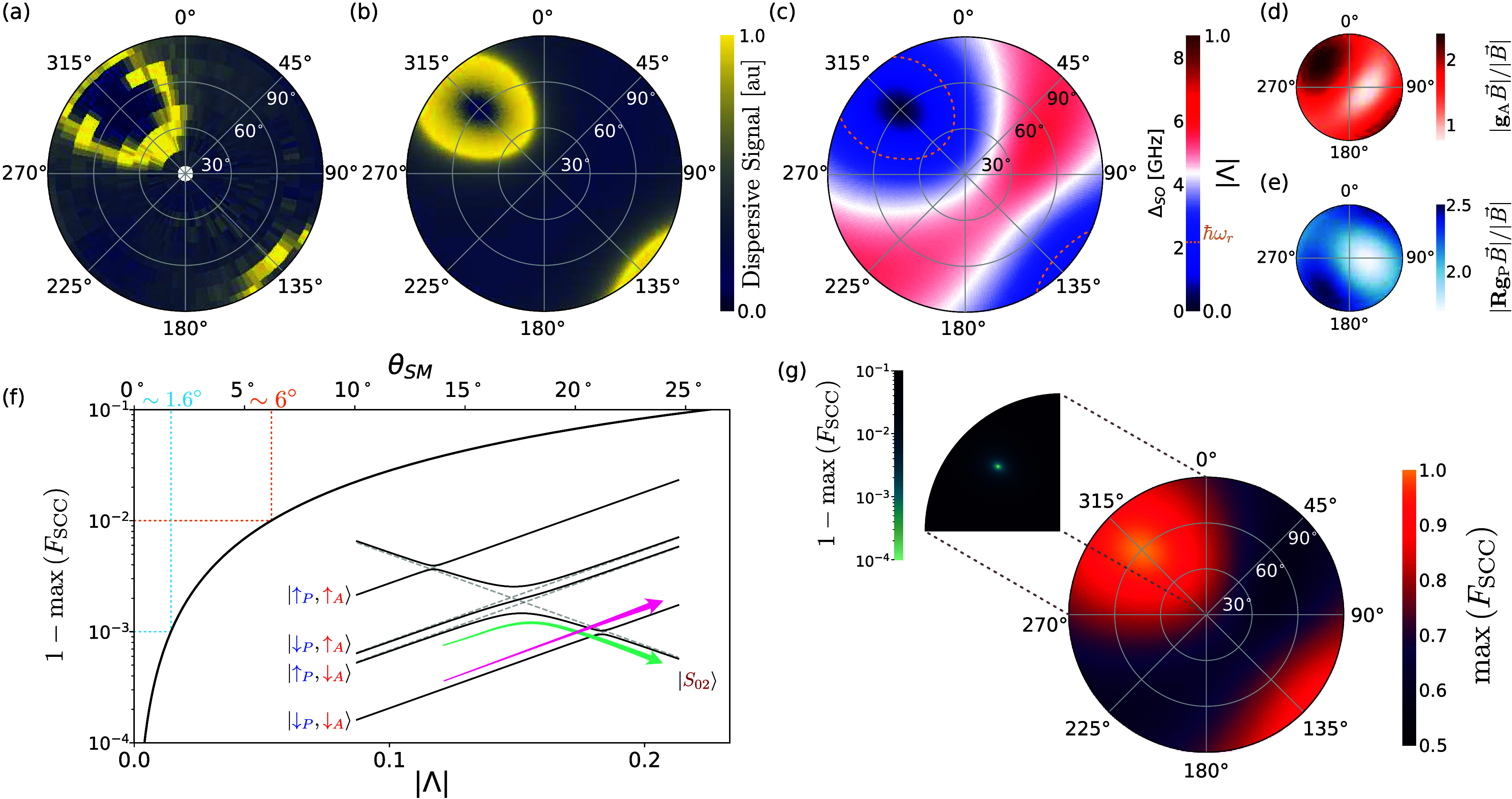
**Spin misalignment and spin readout.** Maximum dispersive signal data (a) and theory (b) of the charge transition for varying magnetic-field orientation, and (c) fitted spin-misalignment parameter |Λ| and spin–orbit gap (Δ_SO_ = √2*t̃*_c_ |Λ|). (d,e) Measured *g*-tensors of the analyzer (d) and polarizer (e) (see Supporting Information 3). (f) Spin-to-charge conversion (SCC) infidelity 1 – *F*_SCC_ (tunneling process in the inset) as a function of spin misalignment. (g) Expected *F*_SCC_ based on the parameters extracted from the data. The inset shows the minimum SCC infidelity on a logarithmic scale around the PSB direction.

To explore this further, we separately characterize the *g*-tensors of the two QDs (Figure 3d,e, see Supporting Information 3). First, we note that the PSB direction closely corresponds to the maximum Zeeman splitting in the analyzer. This is a direct consequence of **g**_A_ having a stronger asymmetry than **Rg**_P_. Similarly, we see the PSB halo *squeezed* longitudinally. This is caused by both tensors having a larger gradient along the equator, resulting in the spins becoming misaligned faster in one direction. *En passant*, we stress that none of the principal axes of the *g*-tensors are aligned with the nanowire, nor do the *g*-tensors themselves respect the symmetry properties expected from the origin of the hole confinement (cylindrical for the QD and spherical for the boron). This fact has already been observed in the literature,^53–56^ and it is testament to how SOC is a strong function of the local electromagnetic environment of each QD, highlighting the need for an accurate three-dimensional characterization without *a priori* assumptions on symmetry.^35,57–59^ To this end, the PSB halo provides a clear signature in the hunt for the blockade, which differentiates between the disappearance of the dispersive signal due to PSB, modifications of the resonator impedance due to the magnetic field strength and/or angle,^60^ and vanishing quantum capacitance as Δ_SO_ grows too large.^39^

Lastly, we answer a pressing question in the literature:^27,39^ does every system with strong SOC possess a *magic angle*? Through the language of polarimetry, we can trivially conclude that the answer is unfortunately *no*, by noting that eq 3 describes a (three-dimensional) generalized eigenvalue problem. Therefore, we are guaranteed that there will be at least one field orientation where **Rg**_P_ *B⃗* ∥ **g**_A_ *B⃗*, but the two quantization directions can be parallel (θ_SM_ = 0) or antiparallel (θ_SM_ = π). In the first case, we have normal PSB, while the second corresponds to an *inverted* spin blockade, where the transition to |*S*_02_⟩ is Pauli-blockaded for the *anti*parallel (1,1) states (|↑_P_, ↓_A_⟩ and |↓_P_, |↑_A_⟩), as opposed to parallel states for the noninverted case, and Δ_SO_ is maximized.^27,29^ This is in stark contrast to (two-dimensional) light polarimetry, which always presents a fully bright and a fully dark orientation.

Finally, we exploit the polarimetry analogy to investigate the consequences of spin misalignment on the readout of spin qubits. Even-charge transitions are particularly interesting for quantum information processing. To this end, state manipulation may be performed in the (effective) (1,1) occupation. Readout is then achieved by attempting to pulse to the (0, 2) charge state. PSB will allow the (charge) transition to happen only if the two spins are in antiparallel states, offering a mechanism for spin-to-charge conversion (SCC).^37,56,61^ This process, however, requires states to safely navigate the energy manifold, traversing the singlet anticrossing adiabatically and Δ_SO_ diabatically via a Landau–Zener (LZ) transition (Figure 3f inset).

SCC is conceptually akin to detecting a photon transmitted through a polarimeter, highlighting the possibility of conversion errors due to imperfect spin alignment. To quantify this effect, we introduce the concept of SCC fidelity (*F*_SCC_ ≤ 1), a correction factor for any PSB-based spin readout, which must be taken into account when considering total readout fidelity, as for thermal effects in Elzerman readout.^62,63^ In particular, *F*_SCC_ sets the fundamental upper limit for the spin-readout fidelity achievable via PSB. Unlike photons (whose speed is constant), *F*_SCC_ strongly depends on the rate of the transition, and its analysis is further complicated by PSB being a nondemolition operation.^56^ A full derivation is presented in the Supporting Information. Nevertheless, for small spin misalignment (θ_SM_ ≲ 10°), the analogy with a polarimeter also holds quantitatively, as it may be shown that max (*F*_SCC_) ≈ 1 – (θ_SM_/2)^2^(1/2 – log|θ_SM_/2|), i.e., the SCC infidelity depends on the square of the misalignment angle, multiplied by a logarithmic correction originating from the LZ formula. Strikingly, this indicates that SCC errors are *inevitable* unless the spins are perfectly aligned (θ_SM_ = 0). It is therefore desirable to perform quantum information processing operations as close as possible to the magic angle. Quantitatively, *F*_SCC_ > 99% requires θ_SM_ ≲ 6°, while *F*_SCC_ > 99.9% requires θ_SM_ ≲ 1.6°, and each additional 9 needs *exponentially* better alignment. We stress that the above is an *upper limit*, which requires pulsing at the optimal ramp rate *ε̇* ∼ *t̃*_c_^2^/ℏ­(see Supporting Information 4). Depending on the system, this may necessitate either pulses beyond the state of the art of signal generation^43^ or entail schemes unacceptably slow considering the ever-increasing requirement of fast spin readout.^37^

The limits above are *universal*, as θ_SM_ is defined in the frame of reference of the internal fields. A conversion to the Cartesian frame of the applied external field requires knowledge of the *g*-tensors of the system. For a concrete example, *F*_SCC_ > 99.9% with the parameters found in this work requires magnetic-field accuracy below 1° (Figure 3g), and would require pulsing in excess of 30 MeV/s.^43^ Notably, *F*_SCC_ may be of concern also for systems where SOC is weak (e.g., electrons in silicon). Spin misalignment due to small differences in *g*-factors may be estimated θ_SM_ ∼ *δg* /*g̅* (*g̅* ∼ 2 being the average *g*-factor). Therefore, the typical tunability of *δg* ∼ 10^–3^ ^64,65^ (up to 10^–2^ ^66–68^ ) could pose challenges to reliably achieving fidelities above 99.9% in large qubit arrays.

In his seminal *Transactions of the Cambridge Philosophical Society*, G. G. Stokes prefaces his analysis of the dynamics of polarized light by stating that “*the object of the philosopher is not to complicate, but to simplify and analyze, so as to reduce phenomena to laws, which in their turn may be made the stepping-stones for ascending to a general theory which shall embrace them all*”.[Fn fn3] In this work, we believe we have upheld the spirit of this statement, for we have reduced the complexity of SOC in spin qubits to a simple law, made familiar by drawing parallels with the ubiquitous technique of polarimetry.

From the analogy with polarimetry naturally arises the definition of the spin misalignment angle, which uniquely determines the avoided crossings in the Hamiltonian of a spin system in the presence of SOC. Importantly for quantum information processing, spin misalignment poses a fundamental upper limit for spin-to-charge conversion via PSB, which forms the basis of many spin-readout schemes. Thus, we have introduced the concept of SCC fidelity, directly linking spin alignment to the achievable fidelity of spin readout. This fundamental limit provides stringent requirements for the accurate and high-resolution characterization of the *g*-tensors and tunnel couplings, highlighting the necessity of precisely identifying the *magic* PSB direction (typically within less than 1°) as a prerequisite to achieving high-fidelity spin readout.

Moreover, a further challenge arises for the scalability of strongly spin–orbit-coupled systems. Particularly, SOC is dependent in the local environment of the spin sites, giving rise to sample-to-sample and device-to-device variability in *g*-tensors.^69^ This translates into strong variability of the spin misalignment and in the *magic* direction of the blockade (if this direction even exists), which may drastically differ even between neighboring QD pairs. Therefore, the limitation of being able to apply *one* global external field may preclude the possibility to perform spin readout on all qubits above the fault-tolerance threshold, putting quantum engineers in the difficult position of deciding between poor-fidelity readout of many QDs or high-quality readout of only a selected few *hero* qubits. We stress that this limit is fundamental in nature, and may only be circumvented if nanofabrication techniques become able to guarantee properties of nominally identical devices down to the defects and local strain in nanostructures. Alternatively, the field may need to advance beyond PSB-based charge sensing, adopting methods such as *in situ* dispersive readout, which can differentiate between spin states even when spin blockade is lifted.^43^

## Supplementary Material



## Data Availability

The data that support the plots within this article and other findings of this study are available at 10.17863/CAM.113242. The unified linear response theory used for the simulations in this work is discussed in ref.^45^ The mathematical equations necessary to perform the simulations and data analysis are discussed in the Supporting Information.

## Footnotes

1 A theory “*the wonderful simplicity of which is such as to bear with it the stamp of truth*”. G.G. Stokes, 1851, ref.^19^

2 Paraphrasing Sir R. Penrose describing Dirac’s famed belt trick, when it comes to spins, a 2π rotation turns the world upside-down.^40–42^

3
*Verbatim*, G.G. Stokes, ref.^19^

## Notes

Details on the theoretical methods, experimental setup, and parameter extraction, Figures S1–S4, Equations S1–S26 (PDF)
