# Supplementary material for: Polarimetry with Spins in the Solid State
Source: Nano Lett. 2025 May 7;25(23):9285–92. doi: 10.1021/acs.nanolett.5c01511 (PMC12164523; doi:10.1021/acs.nanolett.5c01511)
Supplement: Supplementary file 1 [file nl5c01511_si_001.pdf]

# Supporting Information: Polarimetry with Spins in the Solid State

Lorenzo Peri,[*,†,‡] Felix-Ekkehard von Horstig,[†,¶] Sylvain Barraud,[§] Christopher J. B. Ford,[‡] Mónica Benito,[‖] and M. Fernando Gonzalez-Zalba[*,†]

†*Quantum Motion, 9 Sterling Way, London, N7 9HJ, United Kingdom*

‡*Cavendish Laboratory, University of Cambridge, JJ Thomson Ave, Cambridge CB3 0HE, United Kingdom*

¶*Department of Materials Sciences and Metallurgy, University of Cambridge, Charles Babbage Rd, Cambridge CB3 0FS, United Kingdom*

§*CEA, LETI, Minatec Campus, F-38054 Grenoble, France*

‖*Institute of Physics, University of Augsburg, Augsburg, 86159, Germany*

E-mail: lp586@cam.ac.uk; fernando@quantummotion.tech

## SI1: Hamiltonian of a Spin-Orbit-Coupled Double Quantum Dot

The Hamiltonian of a double quantum dot (DQD) is generally made up of three components: (i) detuning ($H_\varepsilon$), (ii) Zeeman ($H_\mathrm{Z}$), and (iii) tunneling ($H_\mathrm{t}$). In the presence of SOC, it is characterized by 17 parameters: the DQD detuning $\varepsilon$, the *scalar* spin-conserving $t_0$ and spin-flip tunnel coupling vector $\vec{t} = (t_x, t_y, t_z)^\mathrm{T}$, and 6 parameters for each $3 \times 3$ $g$-tensors. The requirement of time-reversal symmetry forces $t_0$ and $\vec{t}$ to be real, and the $g$-tensors to be real and symmetric.

To more deeply understand the case of an even transition, as described in this work, we quickly discuss the case of an odd transition ($(1,0) \leftrightarrow (0,1)$), to best highlight the physical origin of the spin-misalignment parameter $\Lambda$. In the literature, spin systems are traditionally modelled by choosing an arbitrary quantization axis (usually $\hat{z}$), causing the Hamiltonian to be highly dense[S1,S2] as it convolves the rotations due to the $g$-tensors and $\vec{t}$. In this work, instead, we choose the more natural basis where the quantization axis of each spin is aligned with the relative internal field, thus making the Zeeman contribution diagonal. In this basis the Hamiltonian reads

$$
\begin{aligned}
H^{\text{odd}} =& H_{\varepsilon}^{\text{odd}} + H_{\text{Z}}^{\text{odd}} + H_{\text{t}}^{\text{odd}} && \text{(S1)}\\
H_{\varepsilon}^{\text{odd}} =& \frac{\varepsilon}{2}\left(\left|\uparrow_{\text{P}}\right\rangle\left\langle\uparrow_{\text{P}}\right| + \left|\downarrow_{\text{P}}\right\rangle\left\langle\downarrow_{\text{P}}\right|\right) && \text{(S2)}\\
& -\frac{\varepsilon}{2}\left(\left|\uparrow_{\text{A}}\right\rangle\left\langle\uparrow_{\text{A}}\right| + \left|\downarrow_{\text{A}}\right\rangle\left\langle\downarrow_{\text{A}}\right|\right)\\
H_{\text{Z}}^{\text{odd}} =& \frac{\mu_{\text{B}}}{2}\left|\mathbf{g}_{\text{P}}\vec{B}\right|\left(\left|\uparrow_{\text{P}}\right\rangle\left\langle\uparrow_{\text{P}}\right| - \left|\downarrow_{\text{P}}\right\rangle\left\langle\downarrow_{\text{P}}\right|\right) && \text{(S3)}\\
& +\frac{\mu_{\text{B}}}{2}\left|\mathbf{g}_{\text{A}}\vec{B}\right|\left(\left|\uparrow_{\text{A}}\right\rangle\left\langle\uparrow_{\text{A}}\right| - \left|\downarrow_{\text{A}}\right\rangle\left\langle\downarrow_{\text{A}}\right|\right)\\
H_{\text{t}}^{\text{odd}} =& \tilde{t}_{\text{c}}\Big(Q\left|\downarrow_{\text{P}}\right\rangle\left\langle\downarrow_{\text{A}}\right| + Q^{*}\left|\uparrow_{\text{P}}\right\rangle\left\langle\uparrow_{\text{A}}\right| && \text{(S4)}\\
& \quad + \Lambda\left|\downarrow_{\text{P}}\right\rangle\left\langle\uparrow_{\text{A}}\right| - \Lambda^{*}\left|\uparrow_{\text{P}}\right\rangle\left\langle\downarrow_{\text{A}}\right|\Big) + h.c.
\end{aligned}
$$

where we point out that in odd transitions the choice of analyzer and polarizer is arbitrary. Above, we have defined

$$
\begin{aligned}
|Q| &= \left|\left\langle\downarrow_{\text{A}}\right|W\left|\downarrow_{\text{P}}\right\rangle\right| = \cos\frac{\theta_{\text{SM}}}{2} && \text{(S5)}\\
|\Lambda| &= \left|\left\langle\uparrow_{\text{A}}\right|W\left|\downarrow_{\text{P}}\right\rangle\right| = \sin\frac{\theta_{\text{SM}}}{2}, && \text{(S6)}
\end{aligned}
$$

which highlights the physical interpretation of $\theta_{\text{SM}}$ as the zenith angle of the image of the polarizer ($W\left|\downarrow_{\text{P}}\right\rangle$) on a Bloch sphere, the axis of which is aligned with the internal field of the analyzer. The phases of $Q$ and $\Lambda$ depend on the choice of gauge for the two Kramers

pairs, particularly on the (independent) choices of the $\hat{x}$ and $\hat{y}$ axes for the two separate Bloch spheres. In particular, it is always possible to find a gauge where both $Q$ and $\Lambda$ are real and positive.

It is interesting to point out how the literature traditionally refers to $\vec{t}$ as *spin-flip* tunneling, as it causes a coupling between states of anti-aligned spins.[S2–S4] However, Eq. (S4) shows how this is somewhat of a misnomer, as a finite $\Lambda$ (and thus spin-flip) may arise even if $\vec{t} = 0$. This fact is promptly understood by noticing that if the two quantization axes are themselves misaligned, there will be a finite projection of the respective anti-aligned spins onto one another even in the absence of any rotation. This highlights the complexity of SOC, as its properties are a (highly nontrivial) combination of *all* the Hamiltonian parameters. In particular, from Eqs. (S5) and (S6) we can see how $|Q|^2 + |\Lambda|^2 = 1$, and their function in the Hamiltonian is to distribute the total (zero-field) tunnel coupling between spin-conserving ($Q$) and spin-flip ($\Lambda$) transitions. Hence, it would perhaps be more appropriate to apply the names spin-conserving and spin-flip parameters to $Q$ and $\Lambda$ rather than to $t_0$ and $\vec{t}$.

Strikingly, the complex behavior induced by the presence of SOC may be described by the same parameters $\Lambda$ and $Q$, which, perhaps even more surprisingly, may be employed to describe also even transitions. As mentioned, in this work we consider the $(1,1) \leftrightarrow (0,2)$ transition, which is well-described using only five states: $|{\uparrow_\mathrm{P}}, {\uparrow_\mathrm{A}}\rangle$, $|{\uparrow_\mathrm{P}}, {\downarrow_\mathrm{A}}\rangle$, $|{\downarrow_\mathrm{P}}, {\uparrow_\mathrm{A}}\rangle$, and $|{\downarrow_\mathrm{P}}, {\downarrow_\mathrm{A}}\rangle$ in the $(1,1)$ region and $|S_{02}\rangle$ in the $(0,2)$ occupation. We neglect any excited triplet arising from higher orbital, or valley states, which in our experimental setup is justified by the use of a boron atom as the (doubly occupied) analyzer. Moreover, we assume the mixing of the light and heavy holes, as well as any hyperfine interaction, to be absorbed in the $g$-tensors and spin-orbit vector of the systems.[S5] As above, we discuss the basis in which

spins are aligned with their respective internal field. In this basis, the Hamiltonian reads

$$H^{\text{even}} = H_\varepsilon^{\text{even}} + H_{\text{Z}}^{\text{even}} + H_{\text{t}}^{\text{even}} \tag{S7}$$

$$H_\varepsilon^{\text{even}} = \frac{\varepsilon}{2}\mathbb{I} - \varepsilon \left|S_{02}\right\rangle \left\langle S_{02}\right| \tag{S8}$$

$$H_{\text{Z}}^{\text{even}} = \tag{S9}$$

$$\frac{\mu_{\text{B}}}{2}\Big( \big(|\mathbf{g}_{\text{P}}\vec{B}| + |\mathbf{g}_{\text{A}}\vec{B}|\big) \left|\uparrow_{\text{P}},\uparrow_{\text{A}}\right\rangle \left\langle\uparrow_{\text{P}},\uparrow_{\text{A}}\right|$$

$$-\big(|\mathbf{g}_{\text{P}}\vec{B}| + |\mathbf{g}_{\text{A}}\vec{B}|\big) \left|\downarrow_{\text{P}},\downarrow_{\text{A}}\right\rangle \left\langle\downarrow_{\text{P}},\downarrow_{\text{A}}\right|$$

$$+\big(|\mathbf{g}_{\text{P}}\vec{B}| - |\mathbf{g}_{\text{A}}\vec{B}|\big) \left|\uparrow_{\text{P}},\downarrow_{\text{A}}\right\rangle \left\langle\uparrow_{\text{P}},\downarrow_{\text{A}}\right|$$

$$-\big(|\mathbf{g}_{\text{P}}\vec{B}| - |\mathbf{g}_{\text{A}}\vec{B}|\big) \left|\downarrow_{\text{P}},\uparrow_{\text{A}}\right\rangle \left\langle\downarrow_{\text{P}},\uparrow_{\text{A}}\right| \Big)$$

$$H_{\text{t}}^{\text{even}} = \tag{S10}$$

$$\frac{\tilde{t}_{\text{c}}}{\sqrt{2}}\big(Q \left|\downarrow_{\text{P}},\uparrow_{\text{A}}\right\rangle \left\langle S_{02}\right| - Q^* \left|\uparrow_{\text{P}},\downarrow_{\text{A}}\right\rangle \left\langle S_{02}\right| \tag{S11}$$

$$+\Lambda \left|\downarrow_{\text{P}},\downarrow_{\text{A}}\right\rangle \left\langle S_{02}\right| + \Lambda^* \left|\uparrow_{\text{P}},\uparrow_{\text{A}}\right\rangle \left\langle S_{02}\right| \big) + h.c.,$$

where $\mathbb{I}$ is the $(5 \times 5)$ identity. Notably, we see how, as for the odd case, both $H_\varepsilon$ and $H_{\text{Z}}$ are diagonal in this basis, while $H_{\text{t}}$ connects the $(0, 2)$ singlet with the $(1, 1)$ states. In particular, recalling the definition of $|S_{02}\rangle$, we can see how also in this case $Q$ describes transitions where the transitioning spin's alignment is conserved, while $\Lambda$ describes transition where spin-flip occurs.

## SI2: Dispersive Sensing of Lifted Blockades

Performing polarimetry with spins requires measuring the dispersive signal of an anticrossing that may vanish (at the PSB direction). Thus, interpretation of the experimental data requires a deep understanding of the dispersive interaction between a resonator and a quantum system. Electrically, the quantum system is effectively in parallel (Fig. 2) with the superconducting resonator. If we assume, as is the case in this work, that the radio-frequency

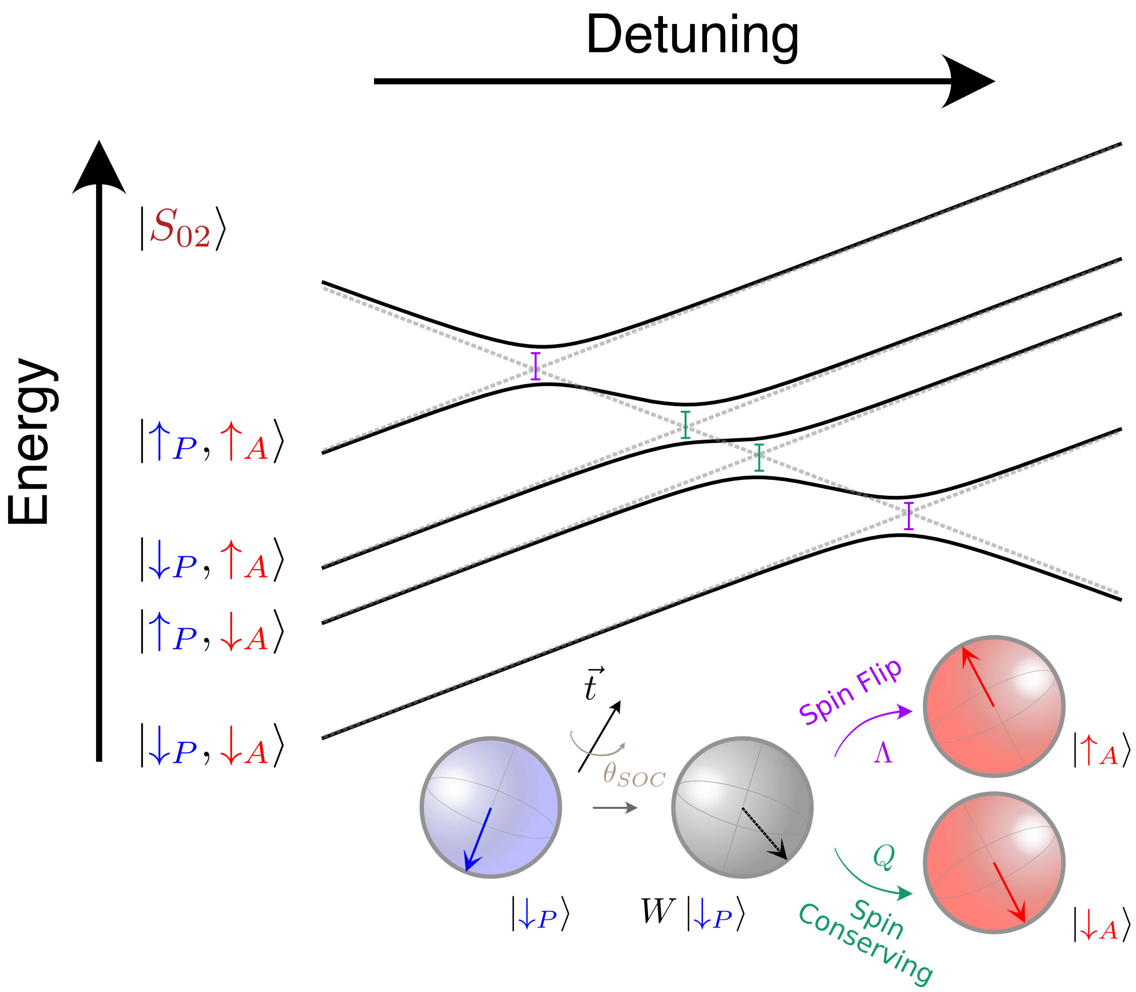


Figure S1: **Energy diagram of a spin-orbit-coupled even-parity transition.** The colors emphasize the effect of $Q$ and $\Lambda$ on the avoided crossing due to spin-flip (purple) and spin-conserving (green) tunneling, pictorially shown in the inset.

excitation through which the system is probed is small enough that the system responds *linearly*, the DQD's behavior may be summarized by a (complex) admittance $Y$.[S6,S7] If we also take, as we shall for simplicity, that the coherent response dominates over the Sisyphus and Hermes components, the admittance reads[S8]

$$Y(\varepsilon) = \frac{\alpha^2 e^2}{\hbar} \sum_{m,n} \frac{(p_m - p_n)\,|\langle\phi_m|\Pi|\phi_n\rangle|^2}{\omega_\mathrm{r} - (E_n - E_m) - i\gamma}, \tag{S12}$$

where $\alpha$ is the (differential) DQD lever arm, $|\phi_m\rangle$ and $E_m$ are the eigenstate and eigenenergy of the Hamiltonian (as a function of $\varepsilon$) occupied with probability $p_m$, $\Pi = \mathrm{d}H/\mathrm{d}\varepsilon$ is the dipole

operator, and $\gamma$ is the decoherence rate. If we assume, as we have in the main text, that the external field is large enough that the DQD may be effectively considered as a two-level system (Eq. (1)), the admittance simplifies to[S8]

$$Y(\varepsilon) = i\frac{\alpha^2 e^2}{2\hbar}\frac{\Delta_{\mathrm{SO}}^2}{\Delta E}\frac{\omega_{\mathrm{r}}}{\Delta E^2 + (\gamma + i\omega_{\mathrm{r}})^2}, \tag{S13}$$

where $\Delta E = \sqrt{\Delta_{\mathrm{SO}}^2 + \varepsilon^2}$ (we assume negligible probability in the excited state). From this we obtain the dispersive signal as the reflection coefficient of a resonator with a variable impedance in parallel,[S9]

$$\Gamma(\omega) \propto \frac{1}{i(\omega - \omega_{\mathrm{r}}) + \kappa/2 + \eta Y}, \tag{S14}$$

where $\kappa$ is the bandwidth of the resonator and $\eta$ is a measure of the (coherent) qubit–resonator coupling.[S10] We note that, for small $\eta$, the changes in reflection coefficient due to changes in the quantum system's admittance (i.e., because of changes in detuning), can be approximated as

$$|\Delta\Gamma| \propto |Y|. \tag{S15}$$

The behavior of Eq. (S13) is shown in Fig. S2, where we see the same trend observed in the main text when discussing Fig. 2. In particular, we see how the admittance, and thus the dispersive signal, increase with decreasing $\Delta_{\mathrm{SO}}$, and the maximum signal is reached when the anticrossing matches the resonator frequency. Intuitively, this originates from the fact that in this regime the resonator is resonantly driving the quantum system, maximizing the response of the charge transition.[S3,S8,S11] If the gap is larger than the photon energy, $|Y|$ takes the form of a single, zero-centered peak with sharply decreasing height proportional to $1/\Delta_{\mathrm{SO}}$. When, instead, $\Delta_{\mathrm{SO}} \leq \hbar\omega_{\mathrm{r}}$, the system responds strongly when the resonance condition is met (red dashed line), and the divergence in the admittance is cured by a finite decoherence rate $\gamma$. As the gap approaches zero (i.e., at the blockade), we see that the signal also vanishes. This is caused by the matrix element of the system's dipole in Eq. (S12), which decreases when the curvature of the energy level diminishes, and vanishes exactly when the

crossing is no longer avoided.

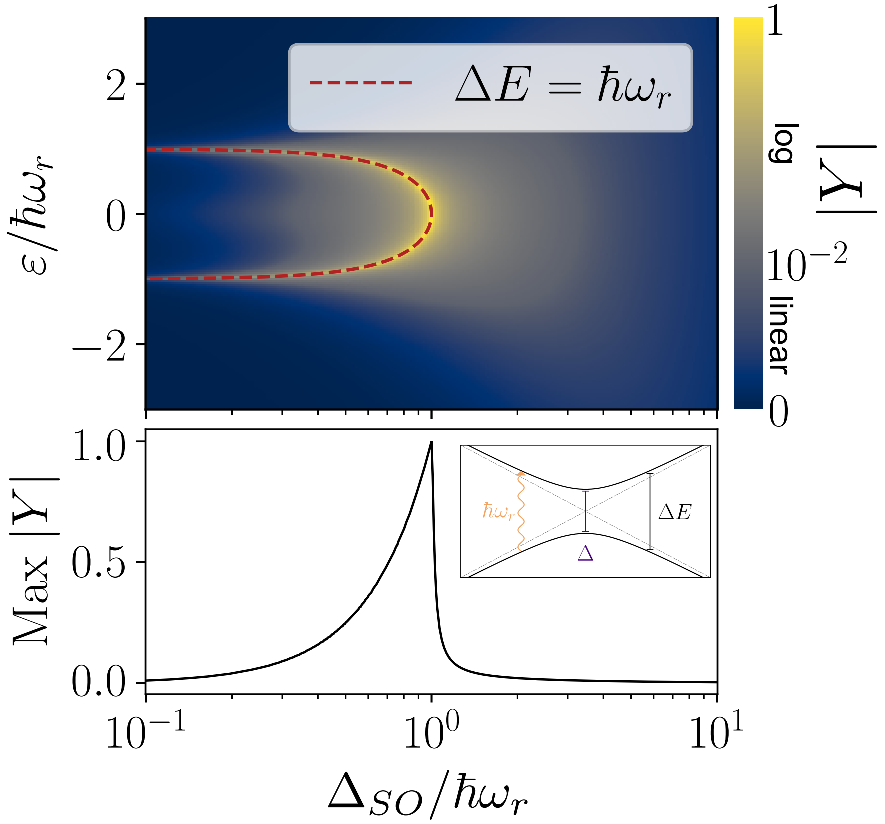


Figure S2: **Dispersive sensing of a lifted blockade.** Absolute value of the equivalent admittance of a two-level (avoided) crossing for varying energy gap ($\Delta_{\mathrm{SO}}$) probed at finite frequency $\omega_{\mathrm{r}}$.

## SI3: Characterization of Physical Parameters

In this work, we separately characterize the $g$-tensors of both QDs, as well as the direction-dependent tunnel coupling, by making use of the visible boron-to-reservoir transition (BRT) adjacent to the inter-dot charge transition (ICT) discussed in the main text. When sweeping the magnetic-field angle, the BRT occurs at $\varepsilon_{\mathrm{BRT}} = \mu_{\mathrm{B}}|\mathbf{g}_{\mathrm{A}}\vec{B}|/2$. Thus, from the slope at various magnetic-field orientations, it is possible to reconstruct $\mathbf{g}_{\mathrm{A}}$ (Fig. S3a-c).

To characterize the ICT parameters, we study the ICT for varying field orientations (Fig. S3d-f). The full-width half-maximum (FWHM) of the dispersive peak is $1.53\Delta_{\mathrm{SO}} \approx$

$2.2|\Lambda|\tilde{t}_c$[S8,S12] (excluding the regions where $\Delta_{SO} < \hbar\omega_r$ and peak-splitting occurs). Fitting these data leads to accurate reconstruction of $|\Lambda|$, and hence we fit $\mathbf{R}\mathbf{g}_P$ (Fig. 3d,e), using the previous knowledge of $\mathbf{g}_A$.

To decouple the rotation due to $\vec{t}$ and $\mathbf{g}_P$, we measure the $(1,1) \leftrightarrow (0,2)$ transition as a function of magnetic field. At high field, the ICT occurs at $\varepsilon_{ICT} = \mu_B(|\mathbf{g}_A\vec{B}| + |\mathbf{g}_P\vec{B}|)/2$. Thus, fitting this slope for various field directions, we derive $|\mathbf{g}_A\vec{B}| + |\mathbf{g}_P\vec{B}|$ (Fig. S3g-i).

The parameters obtained through this process are

$$\mathbf{g}_A = \begin{pmatrix} 1.7 & -0.4 & 0.3 \\ -0.4 & 1.7 & -0.6 \\ 0.3 & -0.6 & 1.1 \end{pmatrix} \tag{S16}$$

$$\mathbf{g}_P = \begin{pmatrix} 2.4 & 0.1 & 0.0 \\ 0.1 & 2.2 & -0.3 \\ 0.0 & -0.3 & 1.9 \end{pmatrix} \tag{S17}$$

$$\vec{t} = \begin{pmatrix} -4.8, 4.7, -3.4 \end{pmatrix}^{\mathrm{T}} \text{ GHz} \tag{S18}$$

$$t_0 = 9.7\,\text{GHz}. \tag{S19}$$

Recording the BRT in the same sweep of the ICT has the added benefit of allowing for accurate calibration of the intensity of the dispersive signal accounting for changes in the superconducting resonator with magnetic field, as the BRT peak height is independent of field direction.[S6]

## SI4: Landau-Zener Transitions and Spin-to-Charge Conversion Fidelity

Readout of spin-qubit states through PSB (whether dispersively or via charge sensing) requires the states to safely traverse several avoided crossings of potentially different mag-

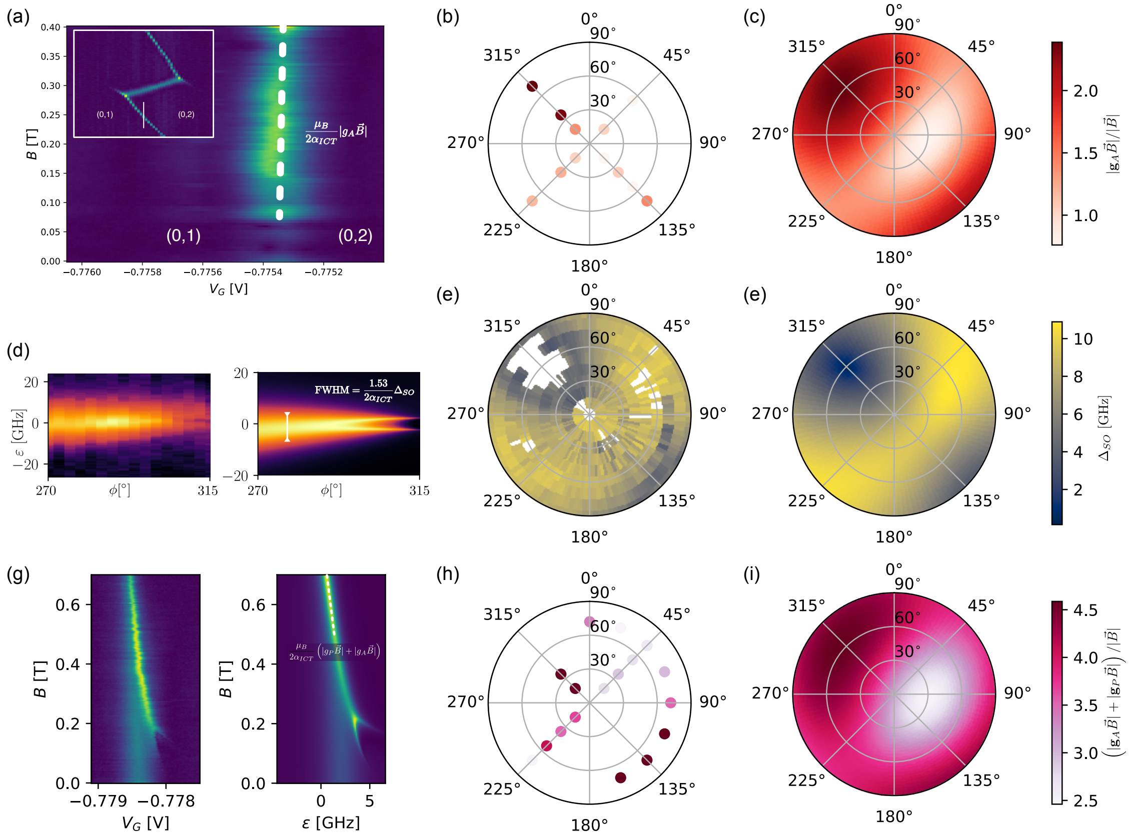


Figure S3: ***g*-tensor and tunnel-coupling characterization** (a-c) Measurement of the $g$-tensor of the boron acceptor (analyzer). The $(1,0) \leftrightarrow (0,2)$ charge transition occurs at $\varepsilon_{\mathrm{BRT}} = \mu_{\mathrm{B}}|\mathbf{g}_{\mathrm{A}}\vec{B}|/2$, thus from its slope at various magnetic field directions (b) it is possible to reconstruct $\mathbf{g}_{\mathrm{A}}$ (c). (d-f) Measurement of $\Delta_{\mathrm{SO}}$. The full-width half-maximum (FWHM) of the dispersive signal from the $(1,1) \leftrightarrow (0,2)$ is $1.53\Delta_{\mathrm{SO}}/2$, from which we fit $\Delta_{\mathrm{SO}} \propto |\Lambda|$. (g-i) To decouple the rotation due to $\vec{t}$ and $\mathbf{g}_{\mathrm{P}}$, we measure the $(1,1) \leftrightarrow (0,2)$ transition as a function of magnetic field (g). At high field, the transition occurs at $\varepsilon_{\mathrm{ICT}} = \mu_{\mathrm{B}}(|\mathbf{g}_{\mathrm{A}}\vec{B}| + |\mathbf{g}_{\mathrm{P}}\vec{B}|)/2$. From its slope in various magnetic-field directions (h) it is possible to reconstruct $|\mathbf{g}_{\mathrm{A}}\vec{B}| + |\mathbf{g}_{\mathrm{P}}\vec{B}|$ (i).

nitudes. We discuss here the simplest case, where the computational basis consists of the ground ($|{\downarrow_{\mathrm{P}}}, {\downarrow_{\mathrm{A}}}\rangle$) and first excited ($|{\uparrow_{\mathrm{P}}}, {\downarrow_{\mathrm{A}}}\rangle$) states, i.e., a spin qubit is the polarizer and the analyzer is an ancilla for readout. The results presented here can can, however, be generalized to any state.

To perform charge readout of these states, one must move the states from the $(1,1)$ occupation where then computation occurs to the $(0,2)$ region, where SCC occurs, as (charge) tunneling of the $|{\downarrow_{\mathrm{P}}}, {\downarrow_{\mathrm{A}}}\rangle$ state is forbidden because of PSB. To successfully perform this

operation, the parallel state must diabatically cross the spin-orbit anticrossing. This spin-flip process (from $T_-$ to $|S_{02}\rangle$) occurs with a probability given by the Landau-Zener formula[S13,S14]

$$P_{sf} = 1 - \exp\left(-4\pi|\Lambda|^2 \frac{\tilde{t}_c^2}{\hbar\dot{\varepsilon}}\right), \tag{S20}$$

where, as in the main text, $\dot{\varepsilon}$ is the detuning ramp rate. This is not, however, the only (coherent) error that can occur. The excited state, in fact, needs to adiabatically traverse the (triple) $|\uparrow_{\mathrm{P}}, \downarrow_{\mathrm{A}}\rangle$-$|\downarrow_{\mathrm{P}}, \uparrow_{\mathrm{A}}\rangle$-$|S_{02}\rangle$ crossing, while also diabatically avoiding a spin flip due to finite $\Delta_{\mathrm{SO}}$. The first probability can be calculated exactly via the Demkov-Ostrovsky model, and reads[S15–S18]

$$P_{ct} = 1 - \exp\left(-8\pi|Q|^2 \frac{\tilde{t}_c^2}{\hbar\dot{\varepsilon}}\right), \tag{S21}$$

which represents the probability of the charge transition. We conclude that the fidelity of the SCC process is

$$F_{\mathrm{SCC}} = \frac{(1 - P_{sf})(1 + P_{ct})}{2}, \tag{S22}$$

which, recalling the fact that $|\Lambda|^2 + |Q|^2 = 1$, can never reach unity unless $|\Lambda| = 0$ (Fig. S4). As a matter of fact, it can be shown that

$$\max(F_{\mathrm{SCC}}) = \frac{1}{1+\zeta/2}\left(\frac{\zeta}{1+\zeta/2}\right)^{\frac{\zeta}{2}}, \tag{S23}$$

where $\zeta = |\Lambda|^2/|Q|^2 = \tan^2(\theta_{\mathrm{SM}}/2)$. This is achieved for a ramp rate

$$\dot{\varepsilon}_{\max} = \frac{\tilde{t}_c^2}{\hbar} \frac{8\pi|Q|^2}{\log\left(\frac{1}{|\Lambda|^2} - \frac{1}{2}\right)}. \tag{S24}$$

In the case of quasi-alignment to the PSB direction ($|\Lambda| \ll 1$), Eq. (S23) can be approximated as

$$1 - \max(F_{\mathrm{SCC}}) \approx |\Lambda|^2 \left(\frac{1}{2} - \log|\Lambda|\right), \tag{S25}$$

which, considering that for small spin-misalignment $|\Lambda| \approx \theta_{\rm SM}/2$, is equivalent to the expression in the main text.

We must point out, however, that, depending on the value of $\tilde{t}_{\rm c}$, obtaining the ramp rate in Eq. (S24) may require pulse rates too fast for the state-of-the-art of waveform generation[S19] or too slow to be acceptable considering the ever-increasing demand of fast spin readout, necessary for quantum error correction. On the topic of ramp rates, unlike what may appear at first glance, faster pulses are not always beneficial. Rather, once the ramp rate exceeds $\dot{\varepsilon}_{\rm max}$, $F_{\rm SCC}$ drops very rapidly, following a universal limit (black dashed line in Fig. S4)

$$F_{\rm SCC}^{\rm fast} = 1 - \frac{1}{2}\exp\left(-8\pi\frac{\tilde{t}_{\rm c}^2}{\hbar\dot{\varepsilon}}\right), \tag{S26}$$

which arises from diabatic transitions at the spin-conserving anticrossing.

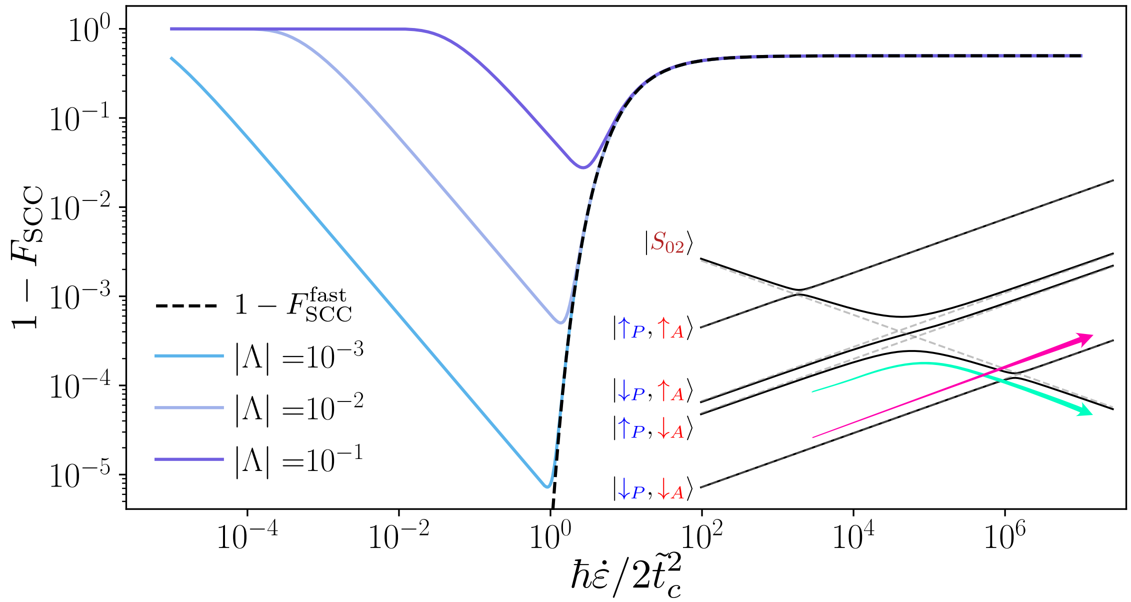


Figure S4: **Spin-to-charge conversion fidelity.** Fidelity of the spin-to-charge conversion process via PSB (inset) for different spin misalignment.

## SI5: Fabrication details

The transistor used in this study is the same as in Ref.[S19] and consists of a single gate silicon-on-insulator (SOI) nanowire transistor with a channel width of 120 nm, a length of 60 nm and height of 8 nm. It was fabricated on an SOI substrate with a 145 nm-thick buried oxide and with a boron doping density of $5 \cdot 10^{17}$ $cm^{-3}$. The silicon layer was patterned to create the channel using optical lithography, followed by a resist trimming process. The transistor gate stack consists of 1.9 nm HfSiON capped by 5 nm TiN and 50 nm polycrystalline silicon, leading to a total equivalent oxide thickness of 1.3 nm. After gate etching, a SiN layer (10 nm) was deposited and etched to form a first spacer on the sidewalls of the gate, then 18-nm-thick Si raised source and drain contacts were selectively grown before source/drain extension implantation and activation annealing. A second spacer was formed, followed by source/drain implantations, an activation spike anneal and silicidation (NiPtSi). The nanowire quantum device and superconducting resonator were connected via on-chip aluminum bond wires.

## SI6: Measurement set-up

Measurements were performed at the base temperature of a dilution refrigerator (T $\sim$ 10 mK). Low-frequency signals ($V_g$, $V_{bg}$) were applied through cryogenic filters, while radio-frequency readout tones were applied through filtered coaxial lines to a coupling capacitor connected to the RF resonator. The resonator consists of a NbTiN superconducting spiral inductor ($L \sim 30\,\mathrm{nH}$), coupling capacitor ($C_c \sim 40\,\mathrm{fF}$) and low-pass filter fabricated by Star Cryoelectronics. For exact details, see Ref.[S20] The PCB was made from 0.8-mm-thick RO4003C with an immersion silver finish. The reflected RF signal was amplified at 4 K and room temperature, followed by quadrature demodulation (Polyphase Microwave AD0540B), from which the amplitude and phase of the reflected signal were obtained (homodyne detection). Magnetic fields were applied using a 5 T/1 T/1 T American Magnetics vector magnet

with the 5 T direction aligned in the plane of the device chip.

## References


(S1) Sen, A.; Frank, G.; Kolok, B.; Danon, J.; Pályi, A. Classification and magic magnetic field directions for spin-orbit-coupled double quantum dots. *Physical Review B* **2023**, *108*, 245406.

(S2) Danon, J.; Nazarov, Y. V. Pauli spin blockade in the presence of strong spin-orbit coupling. *Physical Review B* **2009**, *80*, 041301.

(S3) Benito, M.; Mi, X.; Taylor, J. M.; Petta, J. R.; Burkard, G. Input-output theory for spin-photon coupling in Si double quantum dots. *Physical Review B* **2017**, *96*, 235434.

(S4) Yu, C. X.; Zihlmann, S.; Abadillo-Uriel, J. C.; Michal, V. P.; Rambal, N.; Niebojewski, H.; Bedecarrats, T.; Vinet, M.; Dumur, E.; Filippone, M.; Bertrand, B.; De Franceschi, S.; Niquet, Y.-M.; Maurand, R. Strong coupling between a photon and a hole spin in silicon. *Nature Nanotechnology* **2023**, *18*, 741–746.

(S5) Stano, P.; Loss, D. Quantification of the heavy-hole–light-hole mixing in two-dimensional hole gases. *Physical Review B* **2025**, *111*, 115301.

(S6) Peri, L.; Oakes, G. A.; Cochrane, L.; Ford, C. J. B.; Gonzalez-Zalba, M. F. Beyond-adiabatic Quantum Admittance of a Semiconductor Quantum Dot at High Frequencies: Rethinking Reflectometry as Polaron Dynamics. *Quantum* **2024**, *8*, 1294.

(S7) Oakes, G.; Peri, L.; Cochrane, L.; Martins, F.; Hutin, L.; Bertrand, B.; Vinet, M.; Gomez Saiz, A.; Ford, C.; Smith, C.; Gonzalez-Zalba, M. Quantum Dot-Based Frequency Multiplier. *PRX Quantum* **2023**, *4*, 020346.

(S8) Peri, L.; Benito, M.; Ford, C. J. B.; Gonzalez-Zalba, M. F. Unified linear response theory of quantum electronic circuits. *npj Quantum Information* **2024**, *10*, 1–14.

(S9) Ibberson, D. J.; Lundberg, T.; Haigh, J. A.; Hutin, L.; Bertrand, B.; Barraud, S.; Lee, C.-M.; Stelmashenko, N. A.; Oakes, G. A.; Cochrane, L.; Robinson, J. W.; Vinet, M.; Gonzalez-Zalba, M. F.; Ibberson, L. A. Large Dispersive Interaction between a CMOS Double Quantum Dot and Microwave Photons. *PRX Quantum* **2021**, *2*, 020315.

(S10) Ruskov, R.; Tahan, C. Longitudinal (curvature) couplings of an $N$-level qudit to a superconducting resonator at the adiabatic limit and beyond. *Phys. Rev. B* **2024**, *109*, 245303.

(S11) Kohler, S. Dispersive readout: Universal theory beyond the rotating-wave approximation. *Physical Review A* **2018**, *98*, 023849, Publisher: American Physical Society.

(S12) Esterli, M.; Otxoa, R. M.; Gonzalez-Zalba, M. F. Small-signal equivalent circuit for double quantum dots at low-frequencies. *Applied Physics Letters* **2019**, *114*, 253505.

(S13) Glasbrenner, E. P.; Schleich, W. P. The Landau–Zener formula made simple. *Journal of Physics B: Atomic, Molecular and Optical Physics* **2023**, *56*, 104001.

(S14) Shevchenko, S. N.; Ashhab, S.; Nori, F. Landau-Zener-Stuckelberg interferometry. *Physics Reports* **2010**, *492*, 1–30, arXiv:0911.1917 [cond-mat, physics:physics, physics:quant-ph].

(S15) Band, Y. B.; Avishai, Y. Three-level Landau-Zener dynamics. *Physical Review A* **2019**, *99*, 032112.

(S16) Chernyak, V. Y.; Li, F.; Sun, C.; Sinitsyn, N. A. Integrable multistate Landau-Zener models with parallel energy levels. *Journal of Physics A: Mathematical and Theoretical* **2020**, *53*, 295201, arXiv:2001.10091 [cond-mat, physics:math-ph, physics:quant-ph].

(S17) Demkov, Y. N.; Ostrovsky, V. N. The exact solution of the multistate Landau-Zener

type model: the generalized bow-tie model. *Journal of Physics B: Atomic, Molecular and Optical Physics* **2001**, *34*, 2419.

(S18) Militello, B. Three-state Landau-Zener model in the presence of dissipation. *Physical Review A* **2019**, *99*, 033415.

(S19) von Horstig, F.-E.; Peri, L.; Barraud, S.; Robinson, J. A. W.; Benito, M.; Martins, F.; Gonzalez-Zalba, M. F. Electrical readout of spins in the absence of spin blockade. 2024.

(S20) von Horstig, F.-E.; Ibberson, D. J.; Oakes, G. A.; Cochrane, L.; Wise, D. F.; Stelmashenko, N.; Barraud, S.; Robinson, J. A.; Martins, F.; Gonzalez-Zalba, M. F. Multimodule microwave assembly for fast readout and charge-noise characterization of silicon quantum dots. *Physical Review Applied* **2024**, *21*, 044016.
